# Determinants of adverse birth outcomes among pregnant women in Kintampo municipal hospital, Ghana

**DOI:** 10.3389/fgwh.2025.1444566

**Published:** 2025-03-17

**Authors:** Issah Sumaila, Mubarick Nungbaso Asumah, Mustapha Hallidu, Abraham Ndekudugu, Shaibu Issifu, Anthony Twum, Collins Boateng Danquah, Helen Agodzo, Paulina Clara Appiah, Fred Adomako Boateng

**Affiliations:** ^1^ Ghana Health Service, Kintampo Municipal Hospital, Bono East, Ghana; ^2^ School of Public Health and Allied Science, Catholic University of Ghana, Sunyani, Ghana; ^3^ Ghana Health Service, Kintampo North Municipal Health Directorate, Bono East, Ghana; ^4^ Ghana Health Service, Bono East Regional Health Directorate, Bono East, Ghana

**Keywords:** preterm, stillbirth, low birth weight, congenital abnormality, adverse birth outcome, macrosomia, determinant

## Abstract

**Objective:**

To examine the predictors of adverse birth outcomes (ABOs) among pregnant women attending antenatal clinics at the Kintampo Municipal Hospital (KMH) in Ghana.

**Method:**

A case-control study was conducted to enrol 408 pregnant women attending antenatal clinic at KMH into the study. Structured questionnaire was used to elicit information from the respondents. Stata version 15 was used to analyse the data. Multiple regression analysis was conducted to determine factors associated with ABOs. Level of statistical significance was established at *p* < 0.05.

**Results:**

Factors that were significantly associated with ABOs were: receiving of ITN (aOR = 2.03, 95% CI: 1.20, 3.45), at least 8 times visits to ANC (aOR = 0.32, 95%CI: 0.15, 0.69), and partner's education (aOR = 0.53, 95%CI: 0.29, 0.96).

**Conclusion:**

Contrary to expectations, this study revealed that receiving ITNs during pregnancy was associated with ABOs. Further research is needed to explain why receiving ITNs increases the likelihood of ABOs.

## Introduction

Adverse birth outcomes (ABOs) expressed as low birth weight (LBW); preterm birth (PTB); <37 gestational age at birth, stillbirth, macrosomia, congenital abnormality, abortion and neonatal mortality continue to be a public health issue across the globe especially in developing nations (1–3). According to Wu et al. (4), preterm birth is the second leading cause of death in children below 5 years. When babies are delivered without signs of life at or after 28 weeks of gestation it is described as stillbirth and forms a major component of adverse birth outcomes (5). Adverse birth outcomes remain high in low and middle-income countries (LMICs) because of a multifaceted system of causal factors that include medical, obstetrics and socioeconomic factors (6). The ABO is influenced by genetics and environmental factors such as stunting in early life, maternal health and nutrition, inter-pregnancy intervals, maternal age, genitourinary or general illnesses, and socioeconomic and educational status of women.

Across the world, ABOs affect millions of neonates and nearly one million neonatal mortalities occur every year from preterm births. About fifteen million infants are delivered before thirty-seven weeks of gestation yearly. In Africa and South Asia, greater than 60% of PTB occur, and LBW remains a public health issue across the globe which accounts for 15%–20% of all live births (7). The World Health Organization's (WHO's) worldwide objective for 2025 is to decrease LBW by 30%, that is, a 3% yearly reduction (3). According to a global study on disease burden, the prevalence of stillbirths in LMICs is reported to be 20 per 1,000 live births (8). Nearly 50% of all stillbirths (1.3 million) happened during labor and delivery; and by most current projections, about 15 million infants were delivered as PTB in 2010 representing 11.1% of the entire global live births. Again, more than six out of ten PTB were in South Asia and Sub-Sahara Africa, where more than 50% of the world's live births happen. Additionally, like in other LMICs, available evidence shows that ABO is common in Ethiopia: for example, a current study carried out indicated that almost one out of four (23%) women encounter ABOs (1).

In Africa, the average incidence of LBW babies as a result of ABO is 14.3% (9) and according to the Ghana Demographic Health Survey, 1 out of every 10 newborns in Ghana are LBW (10). Added to the above is a study conducted in Ghana that indicated that the prevalence of ABOs was 19%, and multiparous women were found to be more likely to encounter ABOs (3).

Frequent ANC attendance affords healthcare service providers the chance to identify potential ABOs earlier and manage them. During ANC, different types of pregnancy related interventions such as tetanus diphtheria immunization, malaria prophylaxis, insecticide-treated nets (ITNs) and micronutrient supplements are provided. These interventions have been proven to be effective in managing pregnancy and ABOs (2, 8, 11, 12). Considerable investments in improving maternal and child health will assist in the realization of the Sustainable Development Goals (SDGs) (12).

Ghana provides insecticide-treated nets to pregnant women as a health service to prevent malaria, a leading cause of morbidity and mortality among vulnerable populations. Malaria poses significant health risks to pregnant women, increasing the likelihood of maternal anemia, low birth weight, preterm delivery and maternal and fetal mortality (13, 14). Distributing insecticide-treated nets during antenatal care visits offers convenient access to preventive care, reduces the economic burden on families, and enhances prognosis for mothers and newborns (15, 16).

A 3-year review of the KMH's annual reports from 2019 to 2021 revealed that live births steadily declined respectively over the period (98.7%, 98.5% and 98.1%). The stillbirth rate over the same period increased from 36 to 58 per 1,000 total births over the period. Also, the number of babies born with LBW increased from 90 in 2019 to 113 in 2021 per 1,000 live births. Further, the total number of babies born with LBW increased from 258 in 2019 to 358 in 2021(Kintampo North Municipality DHS, 2022). Adding preterm delivery, congenital birth defects, neonatal mortalities and macrosomia to the list of ABOs in the facility will likely worsen the already bad situation. These indicators undoubtedly show that ABOs are prevalent at the hospital.

Different studies have been conducted on the various forms of ABOs, especially in LMICs and a few parts of Ghana, however, our scholarly search in Kintampo Municipality showed a dearth of health facility-based studies on ABOs. Therefore, this study seeks to identify the predictors of ABOs among pregnant women in KMH.

## Methods

### The study setting

This study was conducted in KMH in the Bono East Region of Ghana.

### Study design and period

This was a case-control study conducted from August 2022 to April 2023.

### Study population

All pregnant women who delivered in KMH during the study period.

### Inclusion criteria

All pregnant women at any stage of their pregnancies who met the following inclusion criteria and provided informed consent were recruited into the study:

i- At least 15 years old and not more than 49 years old
ii- Identified within the study catchment area
iii- Planned to deliver in the study area
iv- Intended to stay in study area until 6 weeks post delivery

Pregnant women who agreed and consented to be part of the study did it for themselves and their unborn babies. All live births by respondents were followed till they were 6 weeks old.

### Exclusion criteria

All pregnant women who failed to provide informed consent were not recruited into the study.

### Description of cases and controls

In this study, the cases were identified as mothers who had one or more ABOs as contained in the operational definition. On the other hand, mothers without ABOs were considered as the control group.

### Sample size determination

The Sample size was calculated using the proportion difference approach using EPI-Info 5.5.8.

A total of 408 (136 cases and 272 controls) respondents were recruited into the study.

All live births in KMH during the study period were assessed based on the operational definition of ABOs.

### Sampling techniques

The participants who qualify to be classified as cases were recruited into the study using a consecutive sampling technique. Also, two participants who qualified to be classified as control were selected consecutively.

### Data collection tools and procedures

A structured questionnaire was developed and adapted for this study after reviewing relevant literature on the subject. Five experienced enumerators were trained for three days on the data collection tool usage and techniques. Thereafter, two days was used for piloting to ensure all enumerators were familiar with all terms and questions. For the cases, a clinical psychologist and a family doctor engaged them to further determine if they have recovered and were in sound mind to participate in the study.

To make sure the participants were free to share their experiences, a separate room was arranged within KMH where all these interviews were conducted face to face. The face-to-face interview approach was adopted because it was thought that most of the study participants were unable to read. To further ensure the right responses were gathered, the questionnaire was translated into Twi and Hausa and read out to the participants. To limit possible interviewer's biases, the data enumerators were persons who could speak and understand Twi and Hausa very well.

### Quality control measures

To ensure consistency, two (2) persons were contacted to translate the questionnaire into the local dialects. Thereafter, two (2) different persons were subsequently contacted to translate the local dialect back to English and then compared with the original questionnaire for consistency.

## Variables

### Dependent variable

The dependent variable was the adverse birth outcomes.

### Operational definition of adverse birth outcomes

The study defined its dependent variable as follows:

Low birth weight (LBW): Live babies who weighed less than 2.5 kg at birth.

Preterm delivery: Live baby born before 37 weeks of gestation.

Congenital birth defects: Physiological or anatomical abnormalities that arise during intrauterine life and discovered prenatally, at birth, or later in infancy, such as hearing problems.

Stillbirths: Death of fetus before birth or at the point of delivery.

Pregnancy loss/miscarriage: Termination of pregnancy before 20 weeks of gestation.

Macrosomia: Baby with a birth weight of more than 4.5 kg at any gestational age of delivery.

Neonatal mortality: Death of a neonate within the first 28 days of life.

### Independent variables

The independent variables included the respondent's socio-demographic characteristics, ANC attendance and services provided and obstetric history.

### Data analysis

The data collected was exported into Microsoft Excel 2019 for cleaning and exported to Stata software version 15 and analysed. Descriptive statistics was subsequently performed on normally distributed continuous variables while proportions were used to present categorical variables. Continuous variables such as maternal age, gravidity, parity, number of ANC visits and gestational age at registration were all categorized. Chi-square analysis was done to find the association between the dependent and the independent variables, and binary and multiple logistic regression analysis done to assess the strength of the associations. Statistically significant factors (*p*-value<0.05) in the binary logistic regression were included in the multiple logistic regression model using the backward stepwise method and analyzed.

### Ethical consideration

The Committee on Human Research, Publication and Ethics (CHRPE) approved the study with clearance ID number *CHRPE/AP/764/22*.

## Results

A total of 408 pregnant women who visited the antenatal clinic at KMH between August 2022 to April 2023 participated in the study. Table 1 provides a summary of the association of sociodemographic characteristics of the women who participated in the study with ABOs. The participants were categorized as cases (137) and controls (271). The mean age of cases (27.9 ± 6.4) and controls (27.9 ± 6.3) were similar. The predictors examined include age, religion, marital status, residence, level of education, and occupation of both the respondents and their partners. The results indicate that there is a statistically significant association between religion (*p* = 0.017), residence (*p* = 0.011) and between partners’ educational level (*p* = 0.007) with ABOs. Specifically, a higher proportion of respondents who were Christians had ABOs compared to those who identified as Muslims or Traditionalists. Additionally, a higher proportion of respondents from rural areas had ABOs compared to those from urban areas. Respondents with partners who had no formal education had a higher proportion of ABOs compared to those with partners who had at least basic education. There were no statistically significant associations between age, marital status, respondents’ educational level, and occupation with ABOs.

**Table 1:** Association of sociodemographic characteristics of respondents with ABOs.

Variables	Cases (137)	Controls (271)	X^2^	*P*-value
	*n* (%)	*n* (%)		
Age			3.69	0.596
15–19	16 (11.7)	23 (8.5)		
20–24	29 (21.1)	75 (27.7)		
25–29	37 (27.0)	62 (22.9)		
30–34	31 (22.7)	65 (24.0)		
35–39	17 (12.4)	36 (13.2)		
≥40	7 (5.1)	10 (3.7)		
Religion			**8**.**17**	**0**.**017**
Christianity	76 (55.4)	181 (66.8)		
Islam	56 (40.9)	88 (32.4)		
Traditional	5 (3.7)	2 (0.8)		
Marital status			1.24	0.743
Single	20 (14.6)	44 (16.2)		
Married	94 (68.6)	182 (67.2)		
Cohabitation	23 (16.8)	43 (15.9)		
Widowed	0 (0)	2 (0.7)		
Residence			**6**.**41**	**0**.**011**
Urban	33 (24.1)	38 (14.0)		
Rural	104 (75.9)	233 (86.0)		
Respondents’ educational level			5.43	0.143
No formal education	43 (31.4)	57 (21.0)		
Basic education	62 (45.2)	138 (50.9)		
Senior high school	20 (14.6)	50 (18.5)		
Tertiary education	12 (8.8)	26 (9.6)		
Partners’ educational level			**12**.**08**	**0**.**007**
No formal education	50 (36.5)	60 (22.2)		
Basic education	31 (22.6	84 (31.0)		
Senior high school	40 (29.2)	76 (28.0)		
Tertiary education	16 (11.7)	51 (18.8)		
Respondents’ occupation			2.55	0.280
Unemployed	58 (42.3)	93 (34.3)		
Self employed	69 (50.4)	157 (57.9)		
Employed	10 (7.3)	21 (7.8)		
Partners’ occupation			1.92	0.383
Unemployed	15 (11.0)	23 (8.4)		
Self employed	102 (74.4)	195 (72.0)		
Employed	20 (14.6)	53 (19.6)		

Bold values are statistically significant at *p* < 0.05.

The association of obstetrics related characteristics with ABOs among the women are summarized in Table 2. The results indicate that gravidity, parity, history of unintended pregnancy, current pregnancy planned, ever used modern contraceptives, modern contraceptive used, and medical history were not significantly associated with ABOs at a 5% significance level. It is important to note that a lack of significant association does not necessarily mean that these predictors are not important factors in predicting ABOs.

**Table 2:** Association of obstetrics related characteristics with ABOs.

Variables	Cases (137)	Controls (271)	X^2^	*P*-value
	*n* (%)	*n* (%)		
Gravidity			1.90	0.388
Primigravida	40 (29.2)	70 (25.8)		
Multigravida	64 (46.7)	146 (53.9)		
Grand multigravida	33 (24.1)	55 (20.3)		
Parity			1.91	0.384
Primiparity	52 (38.0)	85 (31.4)		
Multiparity	64 (46.7)	144 (53.1)		
vGrand multiparity	21 (15.3)	42 (15.5)		
History of unintended pregnancy			0.09	0.764
No	109 (79.6)	223 (82.3)		
Yes	28 (20.4)	48 (17.7)		
Current pregnancy planned (*n* = 76)			0.00	0.972
No	24 (85.7)	41 (85.4)		
Yes	4 (14.3)	7 (14.6)		
Ever used modern contraceptives			2.69	0.101
No	82 (59.9)	139 (51.3)		
Yes	55 (40.1)	132 (48.7)		
Modern contraceptive used			4.29	0.117
Progestin only contraceptive	44 (80.0)	117 (88.6)		
Combined contraceptives	11 (20.0)	13 (9.9)		
Barrier method	0 (0)	2 (1.5)		
Medical history			2.18	0.140
No	119 (86.9)	248 (91.5)		
Yes	18 (13.1)	23 (8.5)		

Table 3 presents the association of history of maternal risky behaviors with adverse birth outcomes. In general, the table shows that there were no significant associations between most of the risky behaviors and adverse birth outcomes, as the *p*-values are greater than 0.05. For instance, the chi-square test results indicate that there was no significant difference between cases and controls with regards to alcohol/alcoholic beverages consumptions, having a history of pica, taking medication without advice of medical personnel, and taking herbal preparations during pregnancy.

**Table 3:** Association of history of maternal risky behaviors with ABOs.

Variables	Cases (137)	Controls (271)	X^2^	*P*-value
	*n* (%)	*n* (%)		
Ever consumed alcohol/alcoholic beverages			1.07	0.301
No	113 (82.5)	234 (86.3)		
Yes	24 (17.5)	37 (13.7)		
Consumed alcohol/alcohol beverage during pregnancy (*n* = 61)			0.57	0.451
No	19 (79.2)	32 (86.5)		
Yes	5 (20.8)	5 (13.5)		
History of pica			0.14	0.707
No	107 (78.1)	216 (79.7)		
Yes	30 (21.9)	55 (20.3)		
Taken medication without advice of medical personnel			0.60	0.437
No	116 (84.7)	237 (87.4)		
Yes	21 (15.3)	34 (12.6)		
Taken herbal preparation during pregnancy			0.06	0.801
No	116 (84.7)	232 (85.6)		
Yes	21 (15.3)	39 (14.4)		

Table 4 provides information on the association of services provided at ANC to reduce the risks of ABOs with ABOs. The results show that attending ANC regularly was not significantly associated with ABOs, with a chi-square test statistic of 3.85 and a *p*-value of 0.050. Women who did not attend ANC regularly were more likely to have ABOs than those who did attend regularly. The number of ANC visits was significantly associated with ABOs, with a chi-square test statistic of 7.06 and a *p*-value of 0.029. Women with fewer than 8 ANC visits were more likely to present with ABOs than those who had at least 8 ANC visits. Other variables, such as gestational age at first ANC visit, consumption of iron and folic acid (IFA) and sulphadoxine-pyrimethamine (SP), and use of insecticide-treated nets (ITNs), were not significantly associated with ABOs.

**Table 4:** Association of services provided at antenatal clinic with ABOs.

Variables	Cases (137)	Controls (271)	X^2^	*P*-value
	n (%)	n (%)		
Attend ANC regularly			3.85	0.050
No	19 (13.9)	21 (7.8)		
Yes	118 (86.1)	250 (92.2)		
Number of ANC visits			**7**.**06**	**0**.**029**
<4 visits	20 (14.6)	18 (6.6)		
4–7 visits	17 (12.4)	32 (11.8)		
≥8 visits	100 (73.0)	221 (81.6)		
Gestational age at first ANC visit			2.15	0.341
First trimester	48 (35.0)	115 (42.5)		
Second trimester	83 (60.6)	144 (53.1)		
Third trimester	6 (4.4)	12 (4.4)		
Given IFA during pregnancy			0.10	0.758
No	7 (5.1)	12 (4.4)		
Yes	130 (94.9)	259 (95.6)		
How often do you consume IFA			4.02	0.134
Daily	109 (83.9)	235 (90.7)		
Sometimes	13 (10.0)	15 (5.8)		
Once in a while	8 (6.1)	9 (3.5)		
Given SP during recent pregnancy			2.18	0.140
No	18 (13.1)	23 (8.5)		
Yes	119 (86.9)	248 (91.5)		
Number SP given during recent pregnancy			2.42	0.120
<3 times	31 (26.0)	47 (19.0)		
≥3 times	88 (74.0)	201 (81.0)		
Did you consume SP			0.13	0.716
No	2 (1.7)	3 (1.2)		
Yes	117 (98.3)	245 (98.8)		
Was all the SP given under DOT			0.27	0.604
No	4 (3.4)	6 (2.4)		
Yes	115 (96.6)	242 (97.6)		
Antihelminth			2.93	0.087
No	37 (27.0)	53 (19.6)		
Yes	100 (73.0)	218 (80.4)		
Given ITNS during pregnancy			**4**.**53**	**0**.**033**
No	4 (3.4)	6 (2.4)		
Yes	115 (96.6)	242 (97.6)		
Sleep in ITNs consistently (*n* = 107)			1.30	0.254
No	37 (27.0)	53 (19.6)		
Yes	100 (73.0)	218 (80.4)		

Bold values are statistically significant at *p* < 0.05.

Table 5 presents the crude and adjusted logistic regression of all predictors associated with ABO in Kintampo Municipal Hospital. The adjusted analysis showed a statistically significant association between three (3) predictors and adverse birth outcome. The predictors were partners’ educational level, number of times women visited ANC during pregnancy and whether or not they were given ITN when they visited ANC.

**Table 5:** Multiple logistic regression analysis for factors associated with birth outcomes.

Variables	Unadjusted		Adjusted	
	OR	95% CI	OR	95% CI
Religion				
Christianity	Ref		Ref	
Islam	1.52	0.99–2.33	1.30	0.82–2.07
Traditional	5.95	1.13–31.36	3.56	0.61–20.86
Residence				
Rural	Ref		Ref	
Urban	0.51	0.31–0.86	0.65	0.37–1.14
Partners’ educational level				
No formal education	Ref		Ref	
Basic education	0.44	0.25–0.77	0.53	0.29–0.96
Senior high school	0.63	0.37–1.07	0.86	0.47–1.55
Tertiary education	0.38	0.19–0.74	0.56	0.27–1.18
Number of ANC visits				
<4 visits	Ref		Ref	
4–7 visits	0.63	0.30–1.31	0.54	0.25–1.19
8 and more	0.33	0.17–0.67	0.32	0.15–0.69
Given ITNS during pregnancy				
No	Ref		Ref	
Yes	1.71	1.04–2.80	2.03	1.20–3.45

After controlling for other predictors at a 95% confidence level, women whose partners had completed basic education were 47% less likely to experience ABO compared with those whose partners had no formal education (aOR = 0.53, 95%CI: 0.29, 0.96).

Women who visited ANC at least 8 times (aOR = 0.32, 95%CI: 0.15, 0.69) were 68% less likely to have an ABO compared with those with less than 4 ANC visits.

After adjusting for other variables, there was 2.0 folds increased odds of ABO in pregnant women who received ITNs compared to those who did not (aOR = 2.03, 95%CI: 1.20, 3.45).

## Discussion

Adverse birth outcomes have consistently remained a global public health problem. This is especially so in LMICs where health infrastructure and policy are a huge challenge. This study found that the pregnant women's partners’ education, number of times the women visited ANC and the women receiving ITNs during pregnancy were the determinants associated with ABOs among pregnant women at the Kintampo municipality hospital.

Pregnant women with partners who have basic education have a 47% reduced risk of adverse birth outcomes, compared to those whose partners have no formal education. This finding is consistent with several other studies that established that pregnant women with educated partner have a reduced risk of adverse birth outcomes compared with those without formal education (3, 17, 18). Though not directly, the educational level of a woman's partner can influence the risk of adverse birth outcomes in several ways including but not limited to economic well-being, support for regular attendance to antenatal care and awareness of danger signs. To begin with, a partner's employment prospects and improved income are often linked to higher education. Easy access to healthcare, nutritious food and healthier living environment are all made possible with improved financial stability, which may result in better birth outcomes. Further, partners with formal education may have a better understanding of the importance of regular antenatal care visits and hence more likely to encourage and support their spouses in attending antenatal care regularly. This may help identify and address any potential complications early on. To conclude, an educated partner is more likely to be aware of the warning signs of potential pregnancy complications. This awareness may lead to timely recognition and seeking medical help, which can improve outcomes in case of emergencies.

As established in this study, women with 8 and more visits to ANC were 68% less likely to have adverse birth outcomes. Consistent with findings from other studies from Ethiopia (1, 2, 19, 20), which revealed that women with at least eight ANC visits are protected from adverse birth outcomes compared with those with less visits. Similarly another study that was conducted in North Wolo Zone, northeast Ethiopia, found that pregnant women with less ANC visit were two times more likely to have adverse birth outcomes compared to those with more antenatal care visits (21). Further, a similar study that was conducted in the Tigrai region, North Ethiopia, established that less than four ANC visits among pregnant women had four times increased odds of ABOs compared with those with more antenatal care visits (1). Regular antenatal care visits have been proven to be important in promoting healthy pregnancy and reducing adverse birth outcomes. It provides the opportunity for pregnant women to receive essential healthcare services and support that reduces the risks of adverse birth outcomes throughout their pregnancy. Also, regular and timely visits to the ANC allow healthcare professionals to monitor the health of both mother and fetus. Through routine tests and screenings, complications can be identified early, allowing for timely interventions and appropriate management. Again, Women who attend ANC regularly are likely to have their nutritional status assessed by healthcare professionals, provided with the needed counseling on four-star diet and appropriate weight gain and receive iron and folic acid tablet supplement. Further, women with history of diseases such as diabetes, hypertension, or infections require specialized care. Thus, regular visits to the ANC help to adequately monitor and manage these conditions effectively, minimizing the risks to both the mother and the baby. In conclusion, pregnant women who regularly visit ANC are more likely to be vaccinated against Td (Tetanus and Diphtheria). All these and many other interventions help in mitigating the risk of adverse birth outcomes among pregnant women.

The study observed that women who received ITNs during pregnancy were twice as likely to have adverse birth outcomes compared with those who did not receive ITNs during pregnancy. This observation is unique and unusual. This is because almost all studies that have been conducted across the globe have found that owning and utilization of ITNs by pregnant women were either significantly associated with reduction in the incidence of adverse birth outcomes, or were not associated with adverse birth outcomes at all (22, 23). This finding could be attributed to the fact that some pregnant women who receive ITNs at ANC do not necessarily sleep under the nets. Some of the reasons they gave for not sleeping under the ITNs were heat, skin irritation, phobia of chemicals used to treat the net and limited airflow under the sleeping space (24–27).

## Conclusion

Determinants such as partner's education level, number of antenatal care visits and receival of ITNs were significantly associated with adverse birth outcomes. Further qualitative studies should be conducted to understand how receiving ITNs predisposed pregnant women to adverse birth outcomes.

## Data Availability

The raw data supporting the conclusions of this article will be made available by the authors, without undue reservation.
